# The role of self-rumination and self-reflection in depressive symptoms among individuals with attention-deficit/hyperactivity disorder traits

**DOI:** 10.1038/s41598-025-88303-x

**Published:** 2025-01-31

**Authors:** Takehiro Tamura, Shunsuke Takagi, Hidehiko Takahashi, Genichi Sugihara

**Affiliations:** 1 Department of Psychiatry and Behavioral Sciences, Graduate School of Medical and Dental Sciences, Institute of Science Tokyo, Tokyo, Japan; 2 Center for Brain Integration Research, Institute of Science Tokyo, Tokyo, Japan; 3 Department of Psychiatry and Behavioral Sciences, Graduate School of Medical and Dental Sciences, Institute of Science Tokyo, 1-5-45 Yushima, Bunkyo-ku, Tokyo, 113-8519 Japan

**Keywords:** Attention-deficit/Hyperactivity disorder (ADHD), Adult ADHD, Depression, Self-rumination, Self-reflection, Mental health, ADHD, Depression, Human behaviour

## Abstract

While some types of rumination have been reported as exacerbating factors in depressive symptoms related to attention-deficit/hyperactivity disorder (ADHD) traits, the role of self-rumination remains underexplored. Additionally, self-reflection—a similar form of private self-consciousness which may have protective effects—has been even less examined. This study of 3,000 Japanese adults (aged 18–50 years) investigates self-rumination and self-reflection in depressive symptoms related to ADHD, using the Adult ADHD Self-Report Scale, Rumination–Reflection Questionnaire, and Beck Depression Inventory-II. Statistical analyses included Pearson’s correlation and linear regression for mediation/moderation. ADHD traits were correlated with depressive symptoms (*r* = 0.413, *p* < 0.001), mediated by self-rumination (indirect effect = 0.21, 95% confidence interval [0.17, 0.26]). Self-reflection was positively correlated with depressive symptoms (*r* = 0.193, *p* < 0.001) but had a protective effect when controlling for self-rumination (β = −0.75, *p* < 0.001). Moderated mediation analysis showed self-reflection reduced depressive symptoms related to ADHD traits by moderating both the impact of ADHD traits on self-rumination and the influence of self-rumination on depressive symptoms. These effects were particularly significant for inattention traits. Our findings suggest that self-reflection, by mitigating self-rumination, may serve as a therapeutic target for depressive symptoms in adults with ADHD traits.

**Supplementary Information:**

The online version contains supplementary material available at 10.1038/s41598-025-88303-x.

## Introduction

Attention-deficit/hyperactivity disorder (ADHD) is a neurodevelopmental disorder characterized not only by attention deficits, hyperactivity, and impulsivity but also by emotional dysregulation. Although some cases of childhood ADHD remit, either spontaneously or with treatment, it often persists into adulthood and is associated with impairments, such as depression^1–3^. Individuals with ADHD exhibit higher rates of depression across childhood, adolescence, and adulthood^4–6^.

A common feature in adults with ADHD is excessive mind-wandering, which can lead to impaired functioning^7^. Mind-wandering, which is considered a universal human experience, diverts attention from external stimuli due to the generation of spontaneous thoughts independent of the immediate environment. One form of this pattern involves focusing on internal thoughts and feelings, which align with the characteristic of private self-consciousness^7–9^.

Based on the distinction between neuroticism and openness to experiences within the five-factor model of personality, private self-consciousness can be divided into two categories, namely self-rumination and self-reflection^8^. On the one hand, self-rumination is defined as the tendency to focus on the self. This tendency is driven by perceived threats, injustice, and loss, and it is directly linked to mental maladjustment by repeatedly focusing on a single point of reference, mainly negative self-related information^8,10,11^. On the other hand, self-reflection is driven by the intellectual curiosity to understand and contemplate the self from an open and accepting perspective^8^. Consequently, this type of private self-consciousness is theoretically regarded as adaptive, and it has been linked to increased levels of self-understanding, autonomy, and mental well-being^8,12^.

Self-rumination, as examined in psychological studies, has consistently been associated with depressive symptoms^12,13^. Conversely, the impact of self-reflection on depression has yielded inconsistent findings, ranging from adaptive to maladaptive effects^10,12–16^. Among the various subtypes of rumination, brooding—a maladaptive pattern of rumination often observed in the context of depression— has been found to mediate the relationship between ADHD and depression, as well as other various risk factors^17,18^. This link between ADHD and brooding may partly stem from the characteristic need for stimulation in individuals with ADHD^19^, which might also contribute to increased self-rumination. However, previous research has not thoroughly examined the relationship between ADHD and private self-consciousness, and thus, the roles of self-rumination and self-reflection in depression related to ADHD remain unclear.

The present study investigates the interrelations among ADHD traits, self-rumination, self-reflection, and depressive symptoms in a Japanese general population of 3,000 individuals aged 18–50 years. Our investigation particularly focuses on the impact of private self-consciousness on the relationship between ADHD traits and depressive symptoms, an area where conclusive findings in the literature are currently lacking. Given the potential protective effects of self-reflection against depression^12^, we hypothesize that self-reflection serves as a crucial protective moderator in the relationship between ADHD traits and depressive symptoms, while self-rumination acts as a key mediator. The aim of this study is to demonstrate these interactions and to elucidate the underlying mechanisms.

## Results

### Sample characteristics

Table 1 shows the descriptive statistics for the entire sample and subgroups categorized by possible ADHD. Participants’ ages spanned from 18 to 50 years (mean age of 36.1 ± 9.4), with 51.9% being woman. The dataset includes participants from all 47 prefectures of Japan, ensuring a geographically diverse sample. While there is a slight overrepresentation of participants from metropolitan areas, this largely reflects the geographic distribution of the population. The distribution of education levels among the 3000 participants, as shown in the pie chart, was predominantly composed of university graduates (*n* = 1447, 48.2%) and high school graduates (*n* = 772, 25.7%) (Figure S1). The possible ADHD group (*n* = 239, 8.0%) showed higher depressive symptoms, self-rumination, and self-reflection compared to the non-ADHD group (*n* = 2,761, 92.0%) (*p* < 0.001). In gender comparisons, self-rumination was higher in women (*p* < 0.001), as opposed to reflection that did not reveal any significant differences (*p* = 0.22) (see Results S1 in Supplementary Material for details).

**Table 1:** Participant characteristics and group comparisons on ADHD traits.

Characteristic	Total (*N* = 3,000)	Possible ADHD (*N* = 239)	Non-ADHD (*N* = 2,761)	*P*-value	Effect size
Age (years) mean (SD)	36.1 (9.4)	32.1 (9.7)	36.4 (9.3)	< 0.001	0.46
Gender (% man)	48.1	42.3	48.6	0.061	0.03
ASRS mean (SD)	8.8 (4.3)	17.6 (2.7)	8.1 (3.5)	< 0.001	2.73
BDI-II mean (SD)	16.1 (12.5)	29.6 (13.7)	15.0 (11.7)	< 0.001	1.24
Rumination score (SD)	38.3 (8.5)	46.1 (8.1)	37.6 (8.2)	< 0.001	1.07
Reflection score (SD)	35.7 (7.0)	38.8 (8.2)	35.4 (6.9)	< 0.001	0.49

Descriptive statistics for the entire sample and for the presence/absence of possible ADHD. The unpaired t-test and the Pearson’s chi-square test were used to compare continuous and categorical variables, respectively, between the two groups.

ADHD, attention-deficit/hyperactivity disorder; ASRS, Adult ADHD Self-Report Scale; BDI-II, Beck Depression Inventory-II; SD, standard deviation.

### Correlations between ADHD traits, severity of depressive symptoms, and self-rumination or self-reflection

**Fig. 1:**
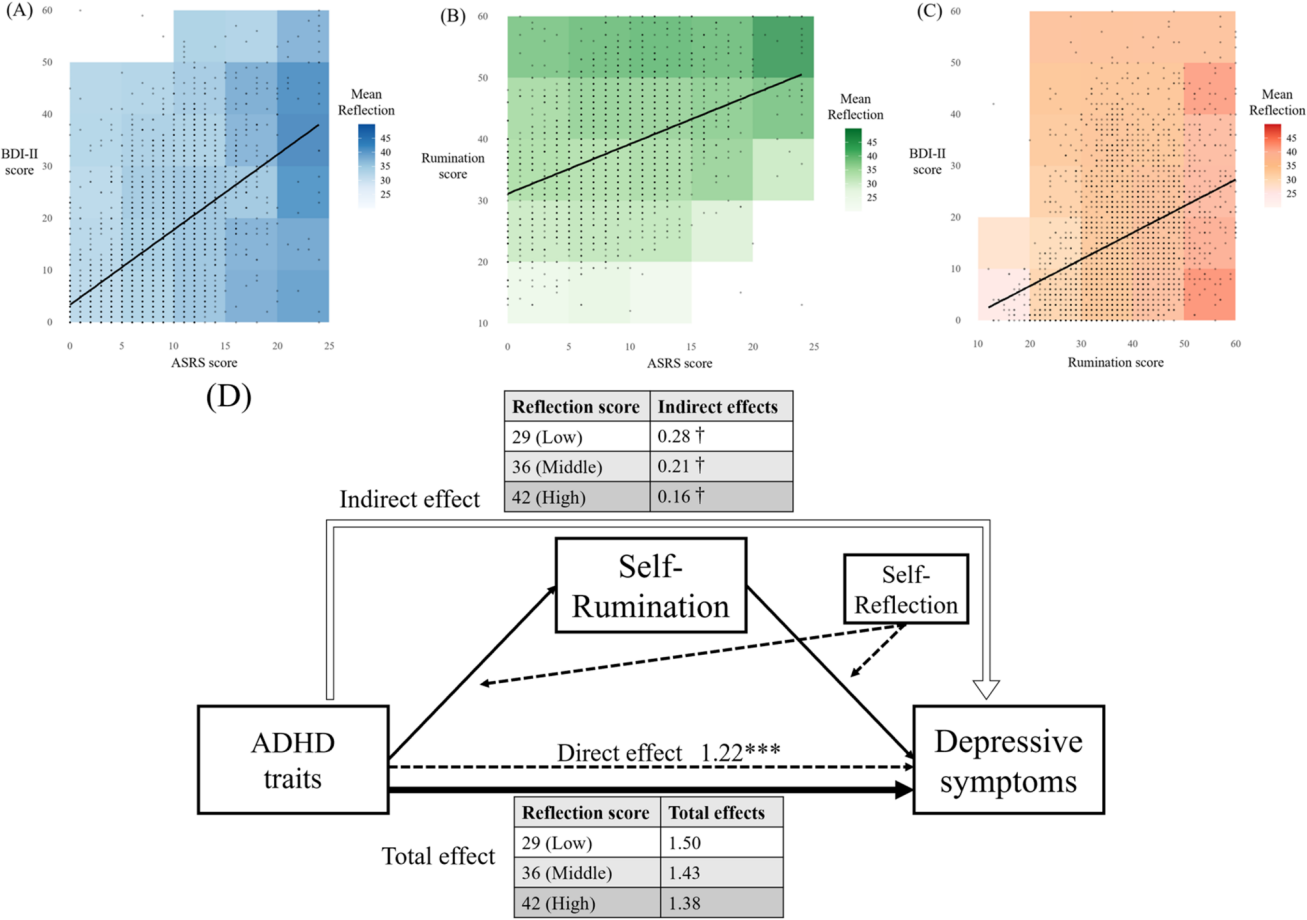
Correlation and moderated mediation patterns of ADHD traits, Self-Rumination, Self-Reflection, and Depressive Symptoms. The background colors in Fig. 1A and B, and 1C illustrate the mean reflection scores. Categories are segmented by ASRS scores (increments of 5), BDI-II scores (increments of 10), and rumination scores (increments of 10). Scores of 60 are included in the 55–59 category, while categories with fewer than three observations are excluded from the average calculation and shown in white.**(A)** Scatter plot showing the correlation between ADHD traits (ASRS score) and depressive symptoms (BDI-II score). Higher ADHD traits are associated with increased depressive symptoms; however, the reflection scores do not exhibit a consistent pattern across these levels.**(B)** Scatter plot showing the correlation between ADHD traits and self-rumination. Self-reflection tends to be linked to both ADHD traits and self-rumination, with a stronger link observed with self-rumination.**(C)** Scatter plot showing the correlation between self-rumination and depressive symptoms (BDI-II score). Contrary to low levels of self-rumination, self-reflection tends to be linked to lower depressive symptoms when self-rumination is high.**(D)** Moderated mediation model illustrating the relationship between ADHD traits (IV) and depressive symptoms (DV), with self-rumination (M) and self-reflection (W). Self-reflection moderates the pathway from IV to M and from M to DV. The direct effect (c’ = 1.22) of ADHD on depressive symptoms along with the indirect effect through self-rumination moderated by self-reflection at different levels (low, middle, high), is shown. Total effects for each level of self-reflection are also presented, demonstrating that self-reflection influences the overall relationship between ADHD traits and depressive symptoms by altering the impact of self-rumination. Furthermore, it is shown that self-reflection significantly reduces the indirect effect of self-rumination on depressive symptoms. † denotes a significant indirect effect.* *p* < 0.001.ADHD, Attention deficit/hyperactivity syndrome; ASRS, ADHD Self-Report Scale; BDI-II, Beck Depression Inventory-II; IV, Independent Variable; DV, Dependent Variable; M, Mediator; W, Moderator.

Pearson correlation analysis revealed significant positive correlations between ADHD traits, depressive symptoms, self-rumination, and self-reflection (Table 2). Specifically, ADHD traits, depressive symptoms, and self-rumination were strongly correlated. These correlations, along with the respective self-reflection score levels, are illustrated in Fig. 1A and C.

**Table 2:** Comprehensive correlation matrix of ADHD traits, depressive symptoms, self-rumination, and self-reflection.

	(1)	(2)	(3)	(4)	Mean (SD)	Range
(1) Total ADHD traits	-				8.83 (4.34)	0–24
(2) Depressive symptoms	0.497 *	-			16.13 (12.53)	0–63
(3) Self-rumination	0.413 *	0.352 *	-		38.26 (8.50)	12–60
(4) Self-reflection	0.193 *	0.085 *	0.368 *	-	35.66 (7.03)	12–60

The correction coefficients were calculated using Pearson’s correlation between two continuous variables. ADHD, attention-deficit/hyperactivity disorder; SD, standard deviations. * *p* < 0.001.

The regression model was significant, F(3, 2996) = 383.55, *p* < 0.001, explaining 27.7% of variance in depressive symptoms, thereby confirming the suitability for mediation analysis (see Results S2 in Supplementary Material for details).

### Indirect and interaction effects of self-rumination and self-reflection on the relationship between ADHD traits and depressive symptoms

We assessed a model proposing that ADHD traits may heighten depressive symptoms, with self-rumination or self-reflection acting as the mediating factor. ADHD traits exhibited a statistically significant direct effect on depressive symptoms, irrespective of the mediating role of self-rumination or self-reflection (self-rumination: 1.22, *p* < 0.001; self-reflection: 1.44, *p* < 0.001). The mediation model through self-rumination indicated a statistically significant indirect effect (0.21, 95% CI [0.17, 0.26]), while the model through reflection did not demonstrate a statistically significant indirect effect (Table S3 in Supplementary Material). Additionally, the examination of whether self-rumination or self-reflection moderated the relationship between ADHD traits and depressive symptoms, showed that neither self-rumination nor self-reflection had a significant moderating effect on this relationship (*p* = 0.64 and 0.34, respectively) (Table S4 in Supplementary Material).

### The impact of ADHD traits on self-rumination and self-reflection

Regression analyses with ADHD traits as the predictor and either self-rumination or self-reflection as the outcome variable demonstrated that only self-rumination could provide significant results. Therefore, we performed mediation and moderation analyses to investigate the pathway from ADHD traits to self-rumination, with self-reflection serving as either a moderating or mediating factor. These analyses revealed that self-reflection acted as both a significant mediator (Indirect effect = 0.11, 95% CI [0.08, 0.13]) and moderator (β = −0.01, *p* = 0.02) in the relationship between ADHD traits and self-rumination. For details, see Results S3 in Supplementary Material.

### Indirect and interaction effects of self-rumination and self-reflection on depressive symptoms

When investigating its role on the relationship between self-rumination and depressive symptoms, self-reflection exhibited a significant moderating effect, indicating that higher levels of self-reflection could mitigate the adverse impact of self-rumination on depressive symptoms (Table 3). In contrast, an investigation of the moderating effect of self-rumination on the relationship between self-reflection and depressive symptoms indicated that self-reflection was not associated with depressive symptoms when self-rumination was low; however, its protective effects against depressive symptoms were amplified when self-rumination was at middle to high levels. This in turn suggests that self-reflection might offer protection against depression in individuals with high self-rumination. The results of our mediation analysis, including the distinct roles of self-reflection and self-rumination as mediators, can be found in the Results S4 in Supplementary Material.

**Table 3:** Interaction effects in moderation analysis: self-reflection and self-rumination in relation to depressive symptoms.

Model	Direct effects of IV on DV		Regression coefficients of W on DV			Regression coefficients of IV x W on DV		
Model 1	0.86 (0.10) ***		0.23 (0.11) *			−0.01 (0.003) **		
Model 2	0.23 (0.11) *		0.86(0.10) ***			−0.01 (0.003) **		
		Conditional effects of the focal predictor at value of the moderator						
Moderator		Condition		Regression coefficients	SE		BCa of 95% CI	
(Model 1)							Lower	Upper
Self-reflection		Low		0.61 ***	0.03		0.55	0.67
		Middle		0.55 ***	0.03		0.5	0.6
		High		0.5 ***	0.03		0.43	0.56
(Model 2)								
Self-rumination		Low		−0.03	0.04		−0.11	0.05
		Middle		−0.10 **	0.03		−0.16	−0.03
		High		−0.17 ***	0.04		−0.25	−0.10

Model (1) IV = Self-rumination, M = Self-reflection, DV = Depression; Model (2) IV = Self-reflection, M = Self-rumination, DV = Depression. All values are unstandardized and reported after controlling for age and sex. BCa represents the bias-corrected and accelerated 95% confidence interval of indirect effects, where intervals not containing 0 indicate a significant indirect effect. * *p* < 0.05, ** *p* < 0.01, *** *p* < 0.001. IV, Independent Variable; DV, Dependent Variable; W, Moderator; CI, Confidence Interval, BCa, Bias-Corrected and Accelerated.

### Moderated mediation analysis of ADHD traits on depressive symptoms

Based on our analyses, we found that self-rumination significantly mediates the relationship between ADHD traits and depressive symptoms, whereas self-reflection does not. Additionally, neither self-rumination nor self-reflection moderated the direct pathway from ADHD traits to depressive symptoms. However, self-reflection did significantly moderate both the relationship between ADHD traits and self-rumination and between self-rumination and depressive symptoms. These findings indicate that the most appropriate model for our study involves self-reflection moderating both the effect of ADHD traits on self-rumination and the effect of self-rumination on depressive symptoms (Figure S2E in Supplementary Material). Self-reflection was a statistically significant moderator of both the path from ADHD traits to self-rumination and the path from self-rumination to depressive symptoms. Subsequently, we assessed the conditional indirect effect using the 16th, 50th, and 84th percentiles of the moderator (Fig. 1D), revealing that the indirect effect was statistically significant at all values. However, higher levels of self-reflection were consistently attenuated the mediating role of self-rumination in the association between ADHD traits and depressive symptoms (Table 4).

**Table 4:** Pathway from ADHD traits to depressive symptoms: Moderated mediation analysis with self-rumination and self-reflection.

Path	IV on M		W on M	IV*W on M		M on DV	W on DV	M*W on DV	IV on DV (Direct effect)
Regression coefficients	1.05 (0.14) ***		0.45 (0.04) ***	−0.01 (0.004) *		0.61 (0.09) ***	0.21 (0.10) *	−0.01 (0.003) ***	1.22 (0.05) ***
		Conditional indirect effects of IV on DV (unstandardized)							
Moderator		Condition			Effect		SE	BCa of 95% CI	
Self-reflection								Lower	Upper
		Low			0.28 †		0.03	0.22	0.33
		Middle			0.21 †		0.02	0.17	0.25
		High			0.16 †		0.03	0.11	0.21

IV = ADHD, M = Self-rumination, DV = Depression, W = Self-reflection.

All values are reported after controlling for age and sex. BCa represents the bias-corrected and accelerated 95% confidence interval of indirect effects. Interval not containing 0 indicates a significant indirect effect. † denotes a significant indirect effect, as the 95% confidence interval does not include zero. * *p* < 0.05, ** *p* < 0.01, *** *p* < 0.001. ADHD, attention-deficit/hyperactivity disorder; IV, Independent Variable; DV, Dependent Variable; M, Mediator; W, Moderator; CI, Confidence Interval, BCa, Bias-Corrected and Accelerated, SE, standard Error.

### Gender differences in the effects of self-rumination and self-reflection in the relationship between ADHD traits and depressive symptoms

To examine potential gender differences, we conducted subgroup analyses stratified by gender (man: *n* = 1442; woman: *n* = 1558). These analyses employed standardized coefficients to allow for direct comparison of effect sizes across subgroups. The results revealed that the indirect effect of ADHD traits on depressive symptoms via self-rumination was significant in both genders (0.07, 95% CI [0.04, 0.09]) and 0.08, 95% CI [0.06, 0.10], respectively). Additionally, self-reflection had a significant protective effect on depressive symptoms and moderated the impact of self-rumination in women, while these effects were not significant in men. For a detailed analysis, see Results S5 in Supplementary Material.

### The role of age in the effects of self-rumination and self-reflection on the relationship between ADHD traits and depressive symptoms

Participants were divided into younger (ages 18–37, *n* = 1562) and older (ages 38–50, *n* = 1438) groups, with the median age of the sample (37 years) included in the younger group. Standardized coefficients were used in the analyses to enable direct comparison of effect sizes across subgroups. The results indicated that the indirect effect of ADHD traits on depressive symptoms via self-rumination was significant in both age groups (younger: 0.06, 95% CI [0.04, 0.07]; older: 0.10, 95% CI [0.07, 0.13]). Additionally, self-reflection showed a significant protective effect on depressive symptoms and moderated the impact of self-rumination in the younger group (interaction effect: -0.05, *p* = 0.005). In contrast, in the older group, self-reflection exhibited a strong direct protective effect on depressive symptoms (standardized coefficient: -0.10, *p* < 0.001), while its moderating effect on the relationship between self-rumination and depressive symptoms was not significant (interaction effect: -0.03, *p* = 0.08). These findings suggest that self-reflection mitigates depressive symptoms through distinct mechanisms depending on age. For a detailed analysis, see Results S6 in Supplementary Material.

### Differential impact of ADHD subscales on depressive symptoms

Our analysis revealed that self-rumination mediates the relationship between ADHD traits and depressive symptoms for both inattention (standardized β = 0.25, *p* < 0.001) and hyperactivity traits (standardized β = 0.23, *p* < 0.001). The standardized indirect effect of inattention traits on depressive symptoms through self-rumination (0.06, 95% CI [0.05, 0.07]) was stronger compared to that of hyperactivity traits (0.02, 95% CI [0.01, 0.03]). Furthermore, the protective effects of self-reflection against depressive symptoms were more pronounced in the relationship between inattention traits and depressive symptoms mediated by self-rumination. These findings suggest that while self-rumination consistently mediates the relationship between ADHD traits and depressive symptoms, this effect is stronger for inattention traits. Additionally, the moderating effects of self-reflection are more significant in the context of inattention traits, providing stronger protective effects against depressive symptoms. For a detailed analysis, including correlation and linear regression analysis, see Results S7 in Supplementary Material.

## Discussion

In this cross-sectional study (*n* = 3,000), we found significant positive correlations among ADHD traits, self-rumination, self-reflection, and depressive symptoms. Furthermore, we observed that self-rumination mediated the relationship between ADHD traits and depressive symptoms, with self-reflection moderating the impact of ADHD traits on self-rumination and the subsequent effect of self-rumination on depressive symptoms. The mediation effect of self-rumination on the relationship between ADHD traits and depressive symptoms was consistently significant regardless of gender or age. Notably, the protective effect of self-reflection on depressive symptoms was prominent in women and exhibited distinct mechanisms depending on age. Furthermore, these associations and effects were more pronounced for inattention traits compared to hyperactivity traits.

Self-rumination demonstrated significant positive correlations with ADHD traits and depressive symptoms, which is in line with the findings of previous studies^13,18,20,21^. Additionally, self-reflection exhibited significant positive correlations with both ADHD traits and depressive symptoms. In contrast to self-rumination, self-reflection was not predicted by ADHD traits and instead showed a negative association with depressive symptoms when controlling for self-rumination. Gender and age differences further highlighted the nuanced role of self-reflection: its protective effect on depressive symptoms was prominent in women and exhibited distinct mechanisms depending on age, with younger individuals showing significant moderating effects and older individuals demonstrating stronger direct effects. These findings suggest that the effects of self-reflection on the relationship between ADHD traits and depressive symptoms are influenced by its interaction with self-rumination, as well as by gender and age. Therefore, considering these interactions is crucial when assessing how self-reflection influences the depressive symptoms related to ADHD traits.

Although previous research suggested the potential protective effects of self-reflection against depressive symptoms^12^, the complex interaction between self-rumination and self-reflection and its effect against depressive symptoms remained unclear. The present study is the first to clarify the interactions among self-rumination, self-reflection, and depressive symptoms through mediation and moderation analyses. While the effect of self-rumination on depressive symptoms is stronger than that of self-reflection, we found that self-reflection serves as a mitigating factor on depressive symptoms by significantly alleviating the impact of self-rumination. This suggests that, although the direct effect of self-reflection on depressive symptoms is relatively small, its influence on self-rumination makes it a meaningful therapeutic target for reducing depressive symptoms.

Additionally, this study revealed for the first time that self-reflection significantly mitigates the relationship between ADHD traits and depressive symptoms. This effect was indirectly achieved by weakening not only the relationship between self-rumination and depressive symptoms but also the relationship between ADHD traits and self-rumination. The mitigating effect of self-reflection on the relationship between ADHD traits and self-rumination is particularly novel. The mechanism driving this effect might be explained by the capacity of self-reflection to enhance metacognitive skills and promote self-understanding, which help individuals recognize and manage attentional biases^8^. Moreover, the indirect effect through self-rumination and the protective effects of self-reflection in the relationship between specific ADHD traits and depressive symptoms were stronger for inattention traits compared to hyperactivity traits. Our results support previous research findings showing that inattention traits were more strongly associated with depression than hyperactivity traits^22^ and suggest that self-rumination may partially explain this difference.

Recent fMRI studies have indicated a common neural basis for ADHD, depression, and rumination, involving dysfunctions in the default mode network (DMN) and the frontoparietal network (FPN)^23–32^. Specifically, FPN dysfunctions are related to difficulties in attention control and emotion regulation, while DMN overactivity leads to persistent self-focus and trouble disengaging from negative thoughts. These studies have revealed that the process of rumination is triggered by excessive salience network activity, exacerbated by impaired frontoparietal network (FPN) function and sustained by excessive default mode network (DMN) activity^28–30^. Although self-rumination, as examined in the present study, is qualitatively different from brooding, often evaluated in fMRI studies, these findings support our understanding of rumination from a transdiagnostic perspective^31,32^. However, the neural basis of self-reflection remains unclear, and further neuroimaging studies are needed to investigate this aspect.

This study has several limitations. First, the cross-sectional design limits our ability to draw causal inferences between ADHD traits, self-rumination, self-reflection, and depressive symptoms. Therefore, longitudinal studies are needed to establish causal pathways more conclusively.

Second, reliance on self-report measures may introduce response biases that are bound to affect the reliability and validity of the acquired data. Third, the present study did not capture healthcare-seeking behaviors, which could provide a more accurate representation of real-world functional impairments. Fourth, this web-based study assessed ADHD traits and depressive symptoms using self-report questionnaires, which may not fully align with clinical diagnoses, potentially affecting the accuracy of our findings. Furthermore, information on additional psychiatric diagnoses and medication use was not collected, meaning the potential influence of comorbid conditions or pharmacological treatment on the observed relationships cannot be ruled out.

Finally, while this study focused on self-rumination, it did not examine the bidirectional relationship between rumination and depression, as shown in prior research using brooding^33^. Additionally, the role of emotional dysregulation—a core ADHD symptom strongly associated with problems in self-concept and functional impairments^34,35^—was not explored. Future studies should investigate how emotional dysregulation interacts with private self-consciousness and depressive symptoms to provide a more comprehensive understanding of these relationships.

Our study highlights the mediating role of self-rumination and the protective moderating effect of self-reflection in the relationship between ADHD traits and depressive symptoms. Interventions targeting both self-rumination and self-reflection may be effective for managing depressive symptoms in individuals with ADHD traits.

## Methods

### Participants

This web-based, cross-sectional study was conducted in February 2024 and recruited participants via Rakuten Insight Inc., in which over two million Japanese individuals have been enrolled. An email with a questionnaire link was sent to individuals all over the country, stratified by district, gender, and age. Participants’ ages ranged from 18 to 50 years. Informed consent was obtained online. Participants were included if they were aged between 18 and 50 years, provided informed consent for this study, and completed the survey in its entirety. Individuals were excluded if they did not provide informed consent, did not complete the survey, or had missing data. We received 3,000 responses that met the inclusion criteria and were used in the subsequent analyses by following the Strengthening the Reporting of Observational Studies in Epidemiology (STROBE) reporting guidelines.

### Setting and procedure

The survey included participants from various urban and rural areas across Japan with the purpose of capturing a diverse range of experiences and backgrounds. The study protocol was approved by the Ethics Committee of Institute of Science Tokyo (formerly Tokyo Medical and Dental University), Tokyo, Japan.

### Measures

ADHD traits were measured using the Japanese version of the Adult ADHD Self-Report Scale (ASRS)^36^, which is based on the diagnostic criteria set by the Diagnostic and Statistical Manual of Mental Disorders, 4th edition, Text Revision (2000)^37^. Participants rated their symptoms over the past 6 months on a five-point Likert scale. Total scores range from 0 to 24, and scores ≥ 15 indicate possible ADHD^36^. The sum of the first four items of ASRS relates to inattention, whereas the last two items to hyperactivity^22^. Depressive symptoms were measured using the Japanese version of Beck Depression Inventory (BDI-II)^38^, with scores ranging from 0 to 63. Self-rumination and self-reflection tendencies were measured using the Japanese version of Rumination–Reflection Questionnaire (RRQ)^39^. The RRQ consists 24 items, divided equally into 12 items for self-rumination and 12 for self-reflection, each rated on a five-point scale. Scores typically range between 12 and 60, with higher scores indicating greater levels of self-rumination or self-reflection. The RRQ is designed to assess relatively stable, trait-like tendencies toward self-focused thought. Unlike state-dependent measures of rumination that primarily capture responses to depressive states, the RRQ evaluates enduring cognitive styles that persist over time and across more diverse contexts^8^.

Participants also provided demographic information including age and gender. Detailed descriptions of these questionnaires are available in the Methods S1 in Supplementary Material.

### Quantitative variables and data preprocessing

ADHD traits, self-rumination, self-reflection, and depressive symptoms were the quantitative variables in this study, all of which were measured on continuous scales. The ASRS, RRQ, and BDI-II are standardized and validated instruments. Data were checked for missing values, and no missing data were found in any of the participant questionnaires.

### Bias

Potential biases were minimized through stratifying participants based on gender and age, using standardized self-report scales, ensuring anonymity, and adjusting for key confounders in the statistical models. Detailed bias mitigation strategies are provided in the Methods S2 in Supplementary Material.

### Statistical analyses

Statistical analyses were performed using SPSS v.28 (IBM Corp., Armonk, NY, USA). First, demographic variables and scores on the ASRS, BDI-II, self-rumination, and self-reflection were compared between potential ADHD and non-ADHD groups, and between genders using t-tests and chi-square tests, with Welch’s t-test applied when necessary.

Second, to ensure the suitability of data for mediation analysis, which requires the independent variable, mediator, and dependent variable to be significantly correlated. Pearson correlation and linear regression analyses were performed to evaluate the relationships between ADHD traits (independent variable), self-rumination (mediator), self-reflection (mediator), and depressive symptoms (independent variable). Detailed descriptions of these linear regression analyses are provided in the Methods S3 in Supplementary Material. All regression analyses controlled for age and gender as covariates.

Third, mediation analysis was used to assess the indirect effect of self-rumination or self-reflection on the relationship between ADHD traits and depressive symptoms, following Baron and Kenny procedure^40^. This mediation model reveals the indirect effect through a mediator and direct effect on the dependent variable. Moderation analysis evaluated whether self-rumination or self-reflection moderates the relationship between ADHD traits and depressive symptoms^41^. All questionnaire scores were treated as continuous variables. Mediation and moderation analyses were performed using the PROCESS macro (v4.2) for SPSS (Figures S2A and S2B in Supplementary Material)^42^. These analyses aimed to investigate mediating and moderating factors that influence the relationship between ADHD traits and depressive symptoms. This statistical tool employs nonparametric resampling procedures (*n* = 5,000) to establish a 95% confidence interval (CI) for the indirect effect. Statistically significant indirect effects are identified by 95% CIs excluding zero. All mediation analyses controlled for age and gender as covariates.

Fourth, mediation and moderation analyses were conducted to evaluate specific relationships within the following two triads (Figures S2A and S2B in Supplementary Material)^42^:

− 1. ADHD traits, self-rumination, and self-reflection.

− 2. Self-rumination, self-reflection, and depressive symptoms.

Detailed descriptions of these analyses are provided in the Methods S4 in Supplementary Material. All moderated mediation analyses controlled for age and gender as covariates.

Finally, we tested our hypothesis using moderated mediation analysis to comprehensively examine the interrelationships between ADHD traits, self-rumination, self-reflection, and depressive symptoms^42–44^. Detailed descriptions of these analyses are provided in the Methods S5 in Supplementary Material.

### Subgroup analysis by gender or age

Given that previous research has indicated higher levels of some types of rumination and depressive symptoms in women compared to men^45,46^, we conducted subgroup analyses stratified by gender to examine whether the relationships between ADHD traits, self-rumination, self-reflection, and depressive symptoms differ between men and women. Mediation and moderated mediation analyses were performed separately for men and women. Age was included as a covariate in all analyses to account for its potential confounding effects.

Similarly, given that previous research has indicated a significant negative correlation between age and self-rumination^47^, we conducted subgroup analyses to examine whether these relationships differ across age groups in adults. Participants were divided into younger and older groups based on the median age. Mediation and moderated mediation analyses were performed separately for each age-based group. Gender was included as a covariate in all analyses to account for its potential confounding effects.

### ADHD subscales analyses

Additional analyses were conducted using ADHD subscales for inattention and hyperactivity traits to explore variations in relationships between variables. This analysis evaluated how relationships between ADHD traits, self-rumination, self-reflection, and depressive symptoms interact differently across these subscales, focusing on the mediating role of self-rumination and the moderating role of self-reflection within each subscale. Standardized coefficients were used to compare these effects across different subscales.

### Handling missing data and sensitivity analyses

All participants had complete data for the primary variables of interest, thus no imputation methods were necessary. Sensitivity analyses were not conducted due to the robustness of the primary analyses and the completeness of the dataset.

The datasets used and/or analyzed during the current study available from the corresponding author upon reasonable request.

## Electronic supplementary material

Below is the link to the electronic supplementary material.

Supplementary Material 1

## Data Availability

The datasets used and/or analyzed during the current study available from the corresponding author upon reasonable request.
