# Supplementary material for: The role of self-rumination and self-reflection in depressive symptoms among individuals with attention-deficit/hyperactivity disorder traits
Source: Sci Rep. 2025 Jan 31;15:3920. doi: 10.1038/s41598-025-88303-x (PMC11785767; doi:10.1038/s41598-025-88303-x)
Supplement: Supplementary file 1 — Supplementary Material 1 [file 41598_2025_88303_MOESM1_ESM.docx]

**Supplementary Material**

**The role of self-rumination and self-reflection in depressive symptoms among individuals with attention-deficit/hyperactivity disorder traits**

**Authors**

**Takehiro Tamura, M.D., Ph.D.; Shunsuke Takagi, M.D., Ph.D.; Hidehiko Takahashi, M.D., Ph.D., and Genichi Sugihara, M.D., Ph.D.**

**Contents**

**Results section:**

**FigureS1, Education levels among 3,000 participants**

**Results S1, Sample characteristics**

**Results S2, Linear regression analysis on depressive symptoms**

**Table S3, Indirect effects of self-rumination or self-reflection in the relationship between ADHD traits and depressive symptoms**

**Table S4, Interaction effects of self-rumination or self-reflection in the relationship between ADHD traits and depressive symptoms**

**Results S3, Impact of ADHD traits on self-rumination and self-reflection: Linear regression, moderation, and mediation analyses**

**Results S4, Indirect effects of self-rumination or self-reflection on depressive symptoms, with interchanged roles of independent and mediator variables**

**Results S5, Gender differences in the effects of self-rumination and self-reflection in the relationship between ADHD traits and depressive symptoms**

**Results S6, The role of age in the effects of self-rumination and self-reflection on the relationship between ADHD traits and depressive symptoms**

**Results S7, Differential impact of ADHD subscales on depressive symptoms**

**Methods section:**

**Methods S2 Detailed descriptions of questionnaires**

**Methods S2 Bias mitigation strategies**

**Methods S3 Linear regression for mediation analysis**

**Figure S2 Schematic diagrams of mediation, moderation, and moderated mediation analyses**

**Methods S4 Mediation and moderation analyses to evaluate specific relationships within two triads**

**Methods S5 Moderated mediation analysis**

**FigureS1 Education levels among 3,000 participants**

**
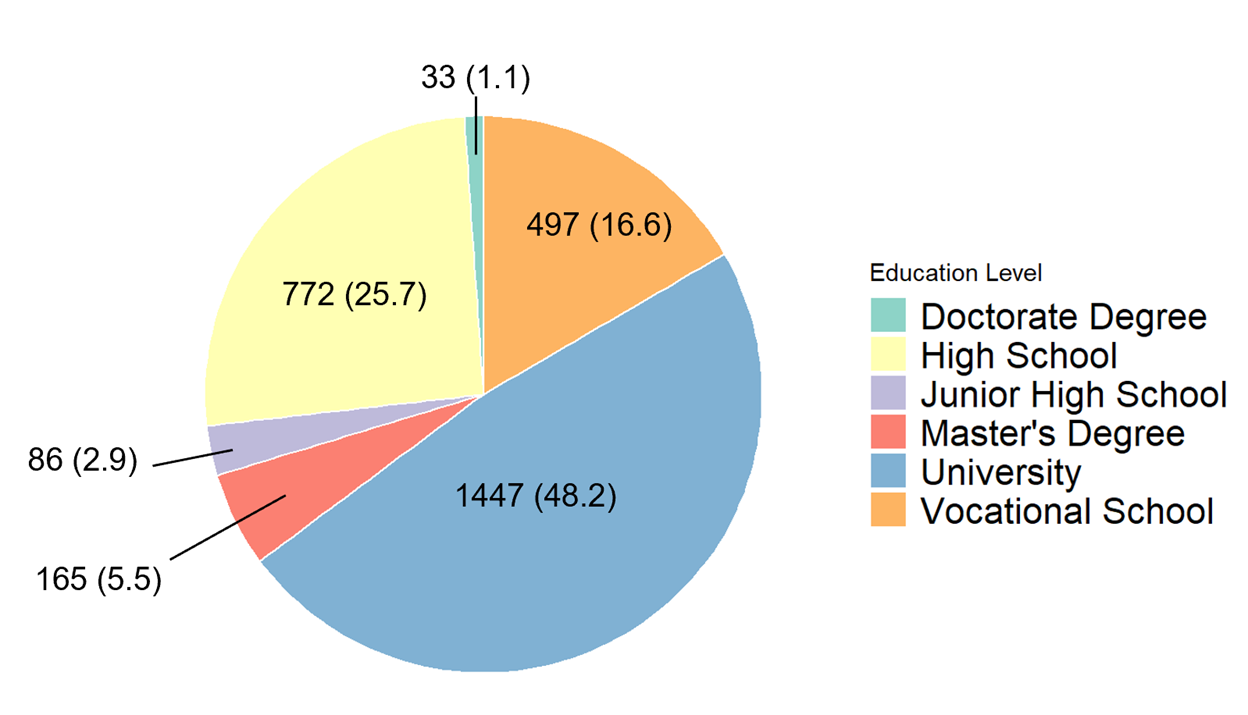
**

The pie chart illustrates the distribution of education levels among 3000 participants, arranged in a clockwise direction starting from the top:

Doctorate Degree: 33 participants (1.1%)

Vocational School: 497 participants (16.6%)

University: 1447 participants (48.2%)

Master's Degree: 165 participants (5.5%)

Junior High School: 86 participants (2.9%)

High School: 772 participants (25.7%)

**Results S1: Sample characteristics**

**(A) Between-group differences in potential ADHD and non-ADHD groups**

There were significant between-group differences with respect to age, severity of depressive symptoms, self-rumination, and self-reflection. The severity of depressive symptoms was significantly increased in the possible ADHD group compared to the non-ADHD group (*p* < 0.001). The percentage of possible depression was 76.2% and 35.5% in the possible ADHD and the non-ADHD groups, respectively. Both self-rumination and self-reflection were significantly increased in the possible ADHD group (*p* < 0.001).

**(B) Between-group differences by gender**

Significant between-group differences were observed in age, self-rumination, and hyperactivity traits. Age was significantly higher in men than in women (*p* < 0.001). Self-rumination levels were significantly higher in women than in man (*p* < 0.001). In contrast, no significant differences were found in ADHD traits, depressive symptoms, and self-reflection between genders (Table S1).

**Table S1 Between-group differences by gender**

| Characteristic | Total  (N = 3,000) | Men  (N = 1,442) | Women  (N = 1,558) | P-value | Effect size |
| --- | --- | --- | --- | --- | --- |
| Age (years)  mean (SD) | 36.1 (9.4) | 38.4 (9.1) | 33.9 (9.2) | <0.001 | 0.49 |
| ASRS  mean (SD) | 8.8 (4.3) | 9.0 (4.3) | 8.7 (4.3) | 0.13 | 0.06 |
| BDI-II  mean (SD) | 16.1 (12.5) | 16.2 (12.7) | 16.1 (12.3) | 0.74 | 0.01 |
| Rumination  score (SD) | 38.3 (8.5) | 37.2 (7.9) | 39.3 (8.9) | <0.001 | 0.25 |
| Reflection  score (SD) | 35.7 (7.0) | 35.8 (6.4) | 35.5 (7.5) | 0.22 | 0.05 |

Descriptive statistics for the entire sample by gender. Unpaired t-test was used to compare continuous variables between the two groups, assuming equal variances. When equal variances could not be assumed, we used the Welch's t-test.

ADHD, attention-deficit/hyperactivity disorder; ASRS, Adult ADHD Self-Report Scale; BDI-II, Beck Depression Inventory-II; SD, standard deviation.

**Results S2: Linear regression analysis on depressive symptoms**

A linear regression analysis, examining factors associated with the severity of depressive symptoms in the participants, revealed a significant association between the severity of depressive symptoms and ADHD traits (β = 0.42, t = 24.51, *p* < 0.001), self-rumination (β = 0.21, t = 11.30, *p* < 0.001), and self-reflection (β = −0.13, t = −4.48, *p* < 0.001). All values are reported after controlling for age and sex. Multicollinearity was assessed in the linear regression analysis. VIF values were under 5, and tolerance values were over 0.2. The results of the linear regression model are presented in Table S2.

**Table S2 Linear regression analysis on depressive symptoms**

|  |  |  |  | Collinearit*y* | |
| --- | --- | --- | --- | --- | --- |
|  | β | t | *p* | Tolerance | VIF |
| ADHD traits | 0.42 | 24.51 | <0.001 | 0.81 | 1.23 |
| Self-rumination | 0.21 | 11.30 | <0.001 | 0.72 | 1.38 |
| Self-reflection | −0.75 | −4.48 | <0.001 | 0.85 | 1.18 |

ADHD, attention-deficit/hyperactivity disorder; VIF, Variance Inflation Factor

**Table S3: Indirect effects of self-rumination or self-reflection in the relationship between ADHD traits and depressive symptoms**

| Model | Regression  coefficients  of IV on M | Regression  coefficients  of M on DV | Direct  effects of  IV on DV | Indirect  effects of  IV on DV | BCa of  95% CI  (Lower) | BCa of  95% CI  (Upper) |
| --- | --- | --- | --- | --- | --- | --- |
| Model  1 | 0.81 (0.03)  *** | 0.26 (0.03)  *** | 1.22  (0.05)  *** | 0.21  (0.02)  † | 0.17 | 0.26 |
| Model  2 | 0.29 (0.03)  *** | −0.02 (0.03) | 1.44  (0.05)  *** | −0.07  (0.01) | −0.02 | 0.01 |

All values are unstandardized and reported after controlling for age and sex. BCa represents the bias-corrected and accelerated 95% confidence interval of indirect effects. Intervals not containing 0 indicate a significant indirect effect. † denotes a significant indirect effect, as the 95% confidence interval does not include zero.

* *p* < 0.05, ** *p* < 0.01, *** *p* < 0.001

Model 1. IV = ADHD, M = Self-rumination, DV = Depression

Model 2. IV = ADHD, M = Self-reflection, DV = Depression

IV, Independent Variable; DV, Dependent Variable; M, Mediator; CI, Confidence Interval, BCa, Bias-Corrected and Accelerated.

**Table S4: Interaction effects of self-rumination or self-reflection in the relationship between ADHD traits and depressive symptoms**

| Model | Direct effects  of IV on DV | Regression  coefficients  of W on DV | Regression  coefficients  of IV*W on DV |
| --- | --- | --- | --- |
| Model 1 | 1.13 (0.18) *** | 0.25 (0.05) *** | 0.002 (0.005) |
| Model 2 | 1.64 (0.22) *** | 0.03(0.06) | −0.006 (0.006) |

All values are unstandardized and reported after controlling for age and sex.

* *p* < 0.05, ** *p* < 0.01, *** *p* < 0.001

Model 1. IV = ADHD, W = Self-rumination, DV = Depression

Model 2. IV = ADHD, W = Self-reflection, DV = Depression

IV, Independent Variable; DV, Dependent Variable; W, Moderator; CI, Confidence Interval, BCa, Bias-Corrected and Accelerated.

**Results S3: Impact of ADHD traits on self-rumination and self-reflection: Linear regression, moderation, and mediation analyses**

A linear regression analysis, examining factors associated with self-rumination in the participants, revealed a significant association between self-rumination and ADHD traits (β = 0.36, t = 22.52, *p* < 0.001) and self-reflection (β = 0.30, t = 18.99, *p* < 0.001). All values are reported after controlling for age and sex. Multicollinearity was assessed in the linear regression analysis. VIF values were under 5, and tolerance values were over 0.2. The results of the linear regression model are presented in Table S5.

**Table S5 Linear regression analysis on self-rumination**

|  |  |  |  | Collinearit*y* | |
| --- | --- | --- | --- | --- | --- |
|  | β | t | *p* | Tolerance | VIF |
| ADHD traits | 0.36 | 22.52 | <0.001 | 0.95 | 1.06 |
| Self-reflection | 0.30 | 18.99 | <0.001 | 0.95 | 1.05 |

ADHD, attention-deficit/hyperactivity disorder; VIF, Variance Inflation Factor

In contrast, a linear regression analysis, examining factors associated with self-reflection in the participants, revealed no significant association between self-reflection and ADHD traits (β = 0.03, t = 1.65, p = 0.100).

Next, we examined the indirect effect of self-reflection on the pathway from ADHD traits to self-rumination. Mediation analysis revealed that self-reflection, functioning as a partial mediator, reduces the direct effect of ADHD traits on self-rumination (Table S6).

**Table S6 Indirect effects of self-reflection in the relationship between ADHD traits and self-rumination**

|  | IV on M | M on DV | IV on DV  (Direct effects) | IV on DV  (Indirect effects) | BCa of 95% CI (Lower–upper) | |
| --- | --- | --- | --- | --- | --- | --- |
| Regression  coefficients | 0.29  (0.03)  *** | 0.37  (0.02)  *** | 0.71 (0.03)  *** | 0.11 (0.01)  † | 0.08 | 0.13 |

Model. IV = ADHD traits, M = Self-reflection, DV = Self-rumination

All values are unstandardized and reported after controlling for age and sex. BCa represents the bias-corrected and accelerated 95% confidence interval of indirect effects. Intervals not containing 0 indicate a significant indirect effect. † denotes a significant indirect effect, as the 95% confidence interval does not include zero.

* *p* < 0.05, ** *p* < 0.01, *** *p* < 0.001

IV, Independent Variable; DV, Dependent Variable; M, Mediator; CI, Confidence Interval, BCa, Bias-Corrected and Accelerated.

We subsequently examined the interaction effect of self-reflection on the pathway from ADHD traits to self-rumination. Self-reflection exhibited a significant moderating effect, indicating that higher levels of self-reflection mitigate the adverse impact of ADHD traits on self-rumination (Table S7).

**Table S7 Interaction effects of self-reflection in the relationship between ADHD traits and self-rumination**

| Model | Direct effects  of IV on DV | Regression  coefficients  of W on DV | | | Regression  coefficients  of IV*W on DV | | |
| --- | --- | --- | --- | --- | --- | --- | --- |
|  | 1.05 (0.14) *** | 0.45 (0.04) *** | | | −0.01 (0.004) * | | |
|  | Conditional effects of the focal predictor at value of the moderator | | | | | | |
| Moderator | Condition | | Regression  coefficients | SE | | BCa of  95% CI | |
|  |  | |  |  | | Lower | Upper |
| Self-reflection | Low | | 0.77 *** | 0.04 | | 0.69 | 0.86 |
|  | Middle | | 0.71 *** | 0.03 | | 0.65 | 0.77 |
|  | High | | 0.65 *** | 0.04 | | 0.58 | 0.73 |

Model. IV = ADHD, W = Self-reflection, DV = Self-rumination

All values are unstandardized and reported after controlling for age and sex. BCa represents the bias-corrected and accelerated 95% confidence interval of indirect effects, where intervals not containing 0 indicate a significant indirect effect.

* *p* < 0.05, ** *p* < 0.01, *** *p* < 0.001, IV, Independent Variable; DV, Dependent Variable; W, Moderator; SE, Standard Error; CI, Confidence Interval, BCa, Bias-Corrected and Accelerated.

**Results S4: Indirect effects of self-rumination or self-reflection on depressive symptoms, with interchanged roles of independent and mediator variables**

Mediation analysis revealed that self-reflection, functioning as a partial mediator, reduces the direct effect of self-rumination on depression (Table S8). When examining the model with self-rumination as a partial mediator, self-rumination was found to completely nullify the protective effect of self-reflection against depression, conversely worsening depressive symptoms.

**Table S8 Indirect effects of self-rumination or self-reflection on depressive symptoms**

| Model | Regression  coefficients  of IV on M | Regression  coefficients  of M on DV | Direct  effects of  IV on DV | Indirect  effects of  IV on DV | BCa of 95% CI (Lower) | BCa of 95% CI (Upper) |
| --- | --- | --- | --- | --- | --- | --- |
| Model  1 | 0.30 (0.01)  *** | −0.11 (0.03)  ** | 0.55 (0.03)  *** | −0.03 (0.01)  † | −0.05 | −0.01 |
| Model  2 | 0.44 (0.02)  *** | 0.55 (0.03)  *** | −0.11 (0.03)  ** | 0.25 (0.02)  † | 0.21 | 0.28 |

Model 1. IV = Self-rumination, M = Self-reflection, DV = Depression

Model 2. IV = Self-reflection, M = Self-rumination, DV = Depression

All values are unstandardized and reported after controlling for age and sex. BCa represents the bias-corrected and accelerated 95% confidence interval of indirect effects. Intervals not containing 0 indicate a significant indirect effect. † denotes a significant indirect effect, as the 95% confidence interval does not include zero.

* *p* < 0.05, ** *p* < 0.01, *** *p* < 0.001

IV, Independent Variable; DV, Dependent Variable; M, Mediator; CI, Confidence Interval, BCa, Bias-Corrected and Accelerated.

**Results S5: Gender differences in the effects of self-rumination and self-reflection in the relationship between ADHD traits and depressive symptoms**

The mediation analysis demonstrated that self-rumination significantly mediated the relationship between ADHD traits and depressive symptoms in both men and women (Table S9 and S10). The indirect effect was significant in men (0.07, 95% CI [0.04, 0.09]) and women (0.08, 95% CI [0.060, 0.101]).

**Table S9 Indirect effects of self-rumination or self-reflection in the relationship between ADHD traits and depressive symptoms in men**

|  | Regression  coefficients  of IV on M | Regression  coefficients  of M on DV | Direct  effects of  IV on DV | Indirect  effects of  IV on DV | BCa of  95% CI  (Lower) | BCa of  95% CI  (Upper) |
| --- | --- | --- | --- | --- | --- | --- |
| Model  1 | 0.42 (0.02)  *** | 0.16 (0.03)  *** | 0.42  (0.03)  *** | 0.07  (0.01)  † | 0.04 | 0.09 |

All values are standardized and reported after controlling for age. BCa represents the bias-corrected and accelerated 95% confidence interval of indirect effects. Intervals not containing 0 indicate a significant indirect effect. † denotes a significant indirect effect, as the 95% confidence interval does not include zero.

* *p* < 0.05, ** *p* < 0.01, *** *p* < 0.001

Model 1. IV = ADHD, M = Self-rumination, DV = Depression

IV, Independent Variable; DV, Dependent Variable; M, Mediator; CI, Confidence Interval, BCa, Bias-Corrected and Accelerated.

**Table S10: Indirect effects of self-rumination or self-reflection in the relationship between ADHD traits and depressive symptoms in women**

| Model | Regression  coefficients  of IV on M | Regression  coefficients  of M on DV | Direct  effects of  IV on DV | Indirect  effects of  IV on DV | BCa of  95% CI  (Lower) | BCa of  95% CI  (Upper) |
| --- | --- | --- | --- | --- | --- | --- |
| Model  1 | 0.41 (0.03)  *** | 0.20 (0.02)  *** | 0.43  (0.02)  *** | 0.08  (0.01)  † | 0.06 | 0.10 |

All values are standardized and reported after controlling for age. BCa represents the bias-corrected and accelerated 95% confidence interval of indirect effects. Intervals not containing 0 indicate a significant indirect effect. † denotes a significant indirect effect, as the 95% confidence interval does not include zero.

* *p* < 0.05, ** *p* < 0.01, *** *p* < 0.001

Model 1. IV = ADHD, M = Self-rumination, DV = Depression

IV, Independent Variable; DV, Dependent Variable; M, Mediator; CI, Confidence Interval, BCa, Bias-Corrected and Accelerated.

The moderated mediation analysis revealed gender differences in the effects of self-reflection on depressive symptoms (Table S11 and S12). In women, self-reflection demonstrated a significant protective effect on depressive symptoms (standardized coefficient: -0.06, *p* = 0.003) and significantly moderated the relationship between self-rumination and depressive symptoms (interaction effect: -0.05, *p* < 0.001). Conditional indirect effects were attenuated at higher levels of self-reflection, with standardized effects of 0.11, 0.08, and 0.05 at low, middle, and high levels of self-reflection, respectively.

In men, while self-reflection also showed a significant direct association with depressive symptoms (standardized coefficient: -0.07, *p* = 0.008), its moderating effect on the relationship between self-rumination and depressive symptoms was not significant (interaction effect: -0.03, *p* = 0.20). Conditional indirect effects for men were smaller overall, with less attenuation across levels of self-reflection (standardized effects: 0.08, 0.07, and 0.06 at low, middle, and high, respectively).

**Table S11: Pathway from ADHD traits to depressive symptoms: Moderated mediation analysis with self-rumination and self-reflection in men**

| Path | IV on M | | W on M | IV*W on M | | M on DV | W on DV | M*W on DV | IV on DV  (Direct effect) |
| --- | --- | --- | --- | --- | --- | --- | --- | --- | --- |
| Regression  coefficients | 0.38 (0.02)  *** | | 0.28 (0.02)  *** | −0.04 (0.02) | | 0.18 (0.03)  *** | -0.07 (0.03)  ** | −0.03 (0.02) | 0.42 (0.03)  *** |
|  | | Conditional indirect effects of IV on DV | | | | | | | |
| Moderator | | Condition | | | Effect | | SE | BCa of 95% CI | |
| Self-reflection | |  | | |  | |  | Lower | Upper |
|  |  | Low | | | 0.08 † | | 0.01 | 0.06 | 0.11 |
|  |  | Middle | | | 0.07 † | | 0.01 | 0.04 | 0.09 |
|  |  | High | | | 0.06 † | | 0.01 | 0.03 | 0.08 |

IV = ADHD, M = Self-rumination, DV = Depression, W = Self-reflection

All values are standardized and reported after controlling for age. BCa represents the bias-corrected and accelerated 95% confidence interval of indirect effects. Interval not containing 0 indicates a significant indirect effect. † denotes a significant indirect effect, as the 95% confidence interval does not include zero.

* *p* < 0.05, ** *p* < 0.01, *** *p* < 0.001

ADHD, attention-deficit/hyperactivity disorder; IV, Independent Variable; DV, Dependent Variable; M, Mediator; W, Moderator; CI, Confidence Interval, BCa, Bias-Corrected and Accelerated, SE, standard Error.

**Table S12: Pathway from ADHD traits to depressive symptoms: Moderated mediation analysis with self-rumination and self-reflection in women**

| Path | IV on M | | W on M | IV*W on M | | M on DV | W on DV | M*W on DV | IV on DV  (Direct effect) |
| --- | --- | --- | --- | --- | --- | --- | --- | --- | --- |
| Regression  coefficients | 0.35 (0.02)  *** | | 0.32 (0.02)  *** | −0.03 (0.02) | | 0.22 (0.02)  *** | -0.06 (0.02)  ** | −0.05 (0.01)  *** | 0.43 (0.02)  *** |
|  | | Conditional indirect effects of IV on DV | | | | | | | |
| Moderator | | Condition | | | Effect | | SE | BCa of 95% CI | |
| Self-reflection | |  | | |  | |  | Lower | Upper |
|  |  | Low | | | 0.11 † | | 0.01 | 0.08 | 0.13 |
|  |  | Middle | | | 0.08 † | | 0.01 | 0.06 | 0.10 |
|  |  | High | | | 0.05 † | | 0.01 | 0.03 | 0.08 |

IV = ADHD, M = Self-rumination, DV = Depression, W = Self-reflection

All values are standardized and reported after controlling for age. BCa represents the bias-corrected and accelerated 95% confidence interval of indirect effects. Interval not containing 0 indicates a significant indirect effect. † denotes a significant indirect effect, as the 95% confidence interval does not include zero.

* *p* < 0.05, ** *p* < 0.01, *** *p* < 0.001

ADHD, attention-deficit/hyperactivity disorder; IV, Independent Variable; DV, Dependent Variable; M, Mediator; W, Moderator; CI, Confidence Interval, BCa, Bias-Corrected and Accelerated, SE, standard Error.

These findings suggest that while self-rumination mediates the relationship between ADHD traits and depressive symptoms in both genders, self-reflection plays a more prominent role in women by not only moderating the impact of self-rumination but also directly reducing depressive symptoms.

**Results S6: The role of age in the effects of self-rumination and self-reflection on the relationship between ADHD traits and depressive symptoms**

The mediation analysis demonstrated that self-rumination significantly mediated the relationship between ADHD traits and depressive symptoms in both men and women (Table S13 and S14). The indirect effect was significant in younger group (0.06, 95% CI [0.04, 0.07]) and older group (0.10, 95% CI [0.07, 0.13]). The strength of this mediating pathway appears to be more pronounced in older adults.

**Table S13 Indirect effects of self-rumination or self-reflection in the relationship between ADHD traits and depressive symptoms in younger group**

|  | Regression  coefficients  of IV on M | Regression  coefficients  of M on DV | Direct  effects of  IV on DV | Indirect  effects of  IV on DV | BCa of  95% CI  (Lower) | BCa of  95% CI  (Upper) |
| --- | --- | --- | --- | --- | --- | --- |
| Model  1 | 0.34 (0.02)  *** | 0.16 (0.03)  *** | 0.40  (0.02)  *** | 0.06  (0.01)  † | 0.04 | 0.07 |

All values are standardized and reported after controlling for sex. BCa represents the bias-corrected and accelerated 95% confidence interval of indirect effects. Intervals not containing 0 indicate a significant indirect effect. † denotes a significant indirect effect, as the 95% confidence interval does not include zero.

* *p* < 0.05, ** *p* < 0.01, *** *p* < 0.001

Model 1. IV = ADHD, M = Self-rumination, DV = Depression

IV, Independent Variable; DV, Dependent Variable; M, Mediator; CI, Confidence Interval, BCa, Bias-Corrected and Accelerated.

**Table S14: Indirect effects of self-rumination or self-reflection in the relationship between ADHD traits and depressive symptoms in older group**

| Model | Regression  coefficients  of IV on M | Regression  coefficients  of M on DV | Direct  effects of  IV on DV | Indirect  effects of  IV on DV | BCa of  95% CI  (Lower) | BCa of  95% CI  (Upper) |
| --- | --- | --- | --- | --- | --- | --- |
| Model  1 | 0.51 (0.03)  *** | 0.19 (0.02)  *** | 0.45  (0.023)  *** | 0.10  (0.01)  † | 0.07 | 0.13 |

All values are standardized and reported after controlling for sex. BCa represents the bias-corrected and accelerated 95% confidence interval of indirect effects. Intervals not containing 0 indicate a significant indirect effect. † denotes a significant indirect effect, as the 95% confidence interval does not include zero.

* *p* < 0.05, ** *p* < 0.01, *** *p* < 0.001

Model 1. IV = ADHD, M = Self-rumination, DV = Depression

IV, Independent Variable; DV, Dependent Variable; M, Mediator; CI, Confidence Interval, BCa, Bias-Corrected and Accelerated.

The moderated mediation analysis revealed age-based differences in the effects of self-reflection on depressive symptoms (Table S15 and S16). In the younger group, self-reflection demonstrated a significant protective effect on depressive symptoms via its moderating role on the relationship between self-rumination and depressive symptoms (interaction effect: -0.05, p = 0.005). In the older group, self-reflection exhibited a strong direct protective effect on depressive symptoms (standardized coefficient: -0.10, p < 0.001), while its moderating effect on the relationship between self-rumination and depressive symptoms was not significant (interaction effect: -0.03, p = 0.08). Conditional indirect effects were attenuated at higher levels of self-reflection in both age groups.

**Table S15: Pathway from ADHD traits to depressive symptoms: Moderated mediation analysis with self-rumination and self-reflection in younger group**

| Path | IV on M | | W on M | IV*W on M | | M on DV | W on DV | M*W on DV | IV on DV  (Direct effect) |
| --- | --- | --- | --- | --- | --- | --- | --- | --- | --- |
| Regression  coefficients | 0.30 (0.02)  *** | | 0.29 (0.02)  *** | −0.02 (0.02) | | 0.19 (0.03)  *** | -0.04 (0.02) | −0.05 (0.02)  ** | 0.40 (0.02)  *** |
|  | | Conditional indirect effects of IV on DV | | | | | | | |
| Moderator | | Condition | | | Effect | | SE | BCa of 95% CI | |
| Self-reflection | |  | | |  | |  | Lower | Upper |
|  |  | Low | | | 0.07 † | | 0.01 | 0.05 | 0.09 |
|  |  | Middle | | | 0.05 † | | 0.01 | 0.04 | 0.07 |
|  |  | High | | | 0.04 † | | 0.01 | 0.02 | 0.06 |

IV = ADHD, M = Self-rumination, DV = Depression, W = Self-reflection

All values are standardized and reported after controlling for sex. BCa represents the bias-corrected and accelerated 95% confidence interval of indirect effects. Interval not containing 0 indicates a significant indirect effect. † denotes a significant indirect effect, as the 95% confidence interval does not include zero.

* *p* < 0.05, ** *p* < 0.01, *** *p* < 0.001

ADHD, attention-deficit/hyperactivity disorder; IV, Independent Variable; DV, Dependent Variable; M, Mediator; W, Moderator; CI, Confidence Interval, BCa, Bias-Corrected and Accelerated, SE, standard Error.

**Table S16: Pathway from ADHD traits to depressive symptoms: Moderated mediation analysis with self-rumination and self-reflection in older group**

| Path | IV on M | | W on M | IV*W on M | | M on DV | W on DV | M*W on DV | IV on DV  (Direct effect) |
| --- | --- | --- | --- | --- | --- | --- | --- | --- | --- |
| Regression  coefficients | 0.45 (0.02)  *** | | 0.31 (0.02)  *** | −0.04 (0.02) | | 0.23 (0.03)  *** | -0.10 (0.02)  *** | −0.03 (0.02) | 0.46 (0.03)  *** |
|  | | Conditional indirect effects of IV on DV | | | | | | | |
| Moderator | | Condition | | | Effect | | SE | BCa of 95% CI | |
| Self-reflection | |  | | |  | |  | Lower | Upper |
|  |  | Low | | | 0.12 † | | 0.03 | 0.09 | 0.15 |
|  |  | Middle | | | 0.10 † | | 0.03 | 0.08 | 0.13 |
|  |  | High | | | 0.09 † | | 0.03 | 0.06 | 0.11 |

IV = ADHD, M = Self-rumination, DV = Depression, W = Self-reflection

All values are standardized and reported after controlling for sex. BCa represents the bias-corrected and accelerated 95% confidence interval of indirect effects. Interval not containing 0 indicates a significant indirect effect. † denotes a significant indirect effect, as the 95% confidence interval does not include zero.

* *p* < 0.05, ** *p* < 0.01, *** *p* < 0.001

ADHD, attention-deficit/hyperactivity disorder; IV, Independent Variable; DV, Dependent Variable; M, Mediator; W, Moderator; CI, Confidence Interval, BCa, Bias-Corrected and Accelerated, SE, standard Error.

These findings suggest that self-reflection attenuates the relationship between ADHD traits and depressive symptoms across age groups, though the mechanisms of this effect differ. In younger individuals, self-reflection primarily moderates the impact of self-rumination, reducing its mediating role. In older individuals, self-reflection exerts a strong direct protective effect on depressive symptoms, which indirectly influences the relationship between ADHD traits and depressive symptoms. This suggests that the protective role of self-reflection extends beyond moderating self-rumination in older individuals.

**Results S7: Differential impact of ADHD subscales on depressive symptoms**

Inattention or Hyperactivity scores also showed significant positive correlations with self-rumination, self-reflection, and severity of depressive symptoms concerning inattention or hyperactivity traits, respectively (Tables S17 and S18).

**Table S17 Comprehensive correlation matrix of inattention traits, depressive symptoms, self-rumination, and self-reflection**

|  | (1) | (2) | (3) | (4) | Mean (SD) | Range |
| --- | --- | --- | --- | --- | --- | --- |
| (1) Inattention traits | - |  |  |  | 6.18 (3.00) | 0–16 |
| (2) Depressive symptoms | 0.457* | - |  |  | 16.13 (12.53) | 0–63 |
| (3) Self-rumination | 0.395* | 0.352* | - |  | 38.26 (8.50) | 12–60 |
| (4) Self-reflection | 0.152* | 0.085* | 0.368* | - | 35.66 (7.03) | 13–60 |

Correction coefficients calculated using Pearson’s correlation between two continuous variables.

* *p* < 0.001, SD, standard deviations

**Table S18 Comprehensive correlation matrix of hyperactivity traits, depressive symptoms, self-rumination, and self-reflection**

|  | (1) | (2) | (3) | (4) | Mean (SD) | Range |
| --- | --- | --- | --- | --- | --- | --- |
| (1) Hyperactivity traits | - |  |  |  | 2.65 (1.82) | 0-8 |
| (2) Depressive symptoms | 0.432* | - |  |  | 16.13 (12.53) | 0-63 |
| (3) Self-rumination | 0.334* | 0.352* | - |  | 38.26 (8.50) | 12-60 |
| (4) Self-reflection | 0.209* | 0.085* | 0.368* | - | 35.66 (7.03) | 13-60 |

Correction coefficients calculated using Pearson's correlation between two continuous variables.

* *p* < 0.001, SD, standard deviation.

In linear regression analyses that included self-rumination, self-reflection, inattention, and hyperactivity traits as independent variables, both inattention (β = 0.25, t = 12.48, *p* < 0.001) and hyperactivity traits (β = 0.23, t = 11.75, *p* < 0.001) were significantly associated with the severity of depressive symptoms as the dependent variable. The results of the linear regression model are presented in Table S19.

**Table S19** **Linear regression analysis on depressive symptoms**

|  |  |  |  | Collinearit*y* | |
| --- | --- | --- | --- | --- | --- |
|  | β | t | *p* | Tolerance | VIF |
| Inattention traits | 0.25 | 12.48 | <0.001 | 0.60 | 1.67 |
| Hyperactivity traits | 0.23 | 11.75 | <0.001 | 0.63 | 1.60 |
| Self-rumination | 0.21 | 11.36 | <0.001 | 0.74 | 1.35 |
| Self-reflection | −0.77 | −4.56 | <0.001 | 0.85 | 1.17 |

VIF, Variance Inflation Factor

In addition, a linear regression analysis, examining factors associated with self-rumination in the participants, revealed a significant association between self-rumination and inattention traits (β = 0.29, t = 14.73, *p* < 0.001) and hyperactivity traits (β = 0.11, t = 5.44, *p* < 0.001). All values are reported after controlling for age and sex. Multicollinearity was assessed in the linear regression analysis. VIF values were under 5, and tolerance values were over 0.2. The results of the linear regression model are presented in Table S20.

**Table S20 Linear regression analysis on self-rumination**

|  |  |  |  | Collinearit*y* | |
| --- | --- | --- | --- | --- | --- |
|  | β | t | *p* | Tolerance | VIF |
| Inattention traits | 0.29 | 14.73 | <0.001 | 0.64 | 1.56 |
| Hyperactivity traits | 0.11 | 5.44 | <0.001 | 0.61 | 1.63 |
| Self-reflection | 0.31 | 19.12 | <0.001 | 0.95 | 1.06 |

ADHD, attention-deficit/hyperactivity disorder; VIF, Variance Inflation Factor

We conducted an analysis using the moderated mediation model (Model 58; Figure S1E) on ADHD traits. Owing to the robust correlation between inattention and hyperactivity traits (r = 0.596, p < 0.001), hyperactivity traits were included as covariates when examining the effects of inattention traits, and vice versa. While the direct effect of self-reflection on depressive symptoms was not significant for either inattention or hyperactivity traits, the interactions in the paths from inattention traits to self-rumination and from self-rumination to depressive symptoms were both significant for inattention traits. In contrast, for hyperactivity traits, the interaction in the path from hyperactivity traits to self-rumination was not significant (Tables S21 and S22).

**Table S21** **Pathway from inattention traits to depressive symptoms: Moderated mediation analysis with self-rumination and self-reflection**

| Path | IV  on M | W  on M | IV*W  on M | M  on DV | W  on DV | M*W  on DV | IV  on DV  (Direct) |
| --- | --- | --- | --- | --- | --- | --- | --- |
| Regression  coefficients | 1.37  (0.21)*** | 0.47  (0.04)*** | −0.02  (0.006)** | 0.61 (0.09)*** | 0.20  (0.10) | −0.01  (0.003)*** | 1.05 (0.08)*** |
|  | Conditional indirect effects of IV on DV (unstandardized) | | | | | | |
| Moderator | Condition | | effect | | SE | BCa of 95% CI | |
| Self-reflection |  | |  | |  | Lower | Upper |
|  | Low | | 0.33 † | | 0.04 | 0.26 | 0.41 |
|  | Middle | | 0.25 † | | 0.03 | 0.19 | 0.31 |
|  | High | | 0.18 † | | 0.03 | 0.12 | 0.24 |

Model. IV = Inattention, M = Self-rumination, DV = Depression, W=Self-reflection

All values are reported after controlling for age, sex and Hyperactivity scores. BCa represents the bias-corrected and accelerated 95% confidence interval of indirect effects. Intervals not containing 0 indicate a significant indirect effect. † denotes a significant indirect effect, as the 95% confidence interval does not include zero.

* *p* < 0.05, ** *p* < 0.01, *** *p* < 0.001, IV, Independent Variable; DV, Dependent Variable; M, Mediator; W, Moderator; SE, Standard Error; CI, Confidence Interval, BCa, Bias-Corrected and Accelerated.

**Table S22 Pathway from hyperactivity traits to depressive symptoms: Moderated mediation analysis with self-rumination and self-reflection**

| Path | IV on  M | W  on M | IV*W  on M | M  on DV | W  on DV | | M*W  on DV | | | IV on DV  (Direct) | |
| --- | --- | --- | --- | --- | --- | --- | --- | --- | --- | --- | --- |
| Regression  coefficients | 0.90  (0.35)* | 0.40  (0.03)*** | −0.01  (0.01) | 0.61  (0.09)*** | 0.20  (0.10) | | −0.01  (0.003)*** | | | 1.56 (0.14)*** | |
|  | Conditional indirect effects of IV on DV (unstandardized) | | | | | | | | | | |
| Moderator | Condition | | | Effect | | SE | | BCa of 95% CI | | | |
| Self-reflection |  | | |  | |  | | | Lower | | Upper |
|  | Low | | | 0.21 † | | 0.04 | | | 0.26 | | 0.41 |
|  | Middle | | | 0.15 † | | 0.03 | | | 0.19 | | 0.31 |
|  | High | | | 0.11 † | | 0.03 | | | 0.12 | | 0.24 |

Model. IV = Hyperactivity, M = Self-rumination, DV = Depression, W = Self-reflection

All values are reported after controlling for age, sex and inattention scores. BCa represents the bias-corrected and accelerated 95% confidence interval of indirect effects. Intervals not containing 0 indicate a significant indirect effect. † denotes a significant indirect effect, as the 95% confidence interval does not include zero.

* *p* < 0.05, ** *p* < 0.01, *** *p* < 0.001, IV, Independent Variable; DV, Dependent Variable; M, Mediator; W, Moderator; SE, Standard Error; CI, Confidence Interval, BCa, Bias-Corrected and Accelerated.

These results suggest that the protective effect of self-reflection against depressive symptoms, mediated by self-rumination, may be more important in the context of inattention traits. Therefore, we calculated the standardized coefficients for each path to evaluate the relative strength of the effects, and also examined how these two effects, namely the mediation effect of self-rumination and the moderation effect of self-reflection, differ between inattention and hyperactivity traits. (Tables S23 and S24)

**Table S23 Pathway from inattention traits to depressive symptoms: Moderated mediation analysis with self-rumination and self-reflection**

| Path | IV  on M | | IV*W  on M | M  on DV | M*W  on DV | | IV on DV  (Direct) | | | |
| --- | --- | --- | --- | --- | --- | --- | --- | --- | --- | --- |
| Regression  coefficients  (standardized) | 0.29  (0.02)*** | | −0.04  (0.01)** | 0.21 (0.02)*** | −0.04  (0.01)*** | | 0.25  (0.02)*** | | | |
|  | | Conditional indirect effects of IV on DV (standardized) | | | | | | | | |
| Moderator | | Condition | | Effect | | SE | | BCa of 95% CI | | |
| Self-reflection | |  | |  | |  | | | Lower | Upper |
|  |  | Low | | 0.08 † | | 0.01 | | | 0.06 | 0.10 |
|  |  | Middle | | 0.06 † | | 0.01 | | | 0.05 | 0.07 |
|  |  | High | | 0.04 † | | 0.01 | | | 0.03 | 0.06 |

Model. IV = Inattention, M = Self-rumination, DV = Depression, W = Self-reflection

All values are reported after controlling for age, sex and Hyperactivity scores. BCa represents the bias-corrected and accelerated 95% confidence interval of indirect effects. Intervals not containing 0 indicate a significant indirect effect. † denotes a significant indirect effect, as the 95% confidence interval does not include zero.

* *p* < 0.05, ** *p* < 0.01, *** *p* < 0.001, IV, Independent Variable; DV, Dependent Variable; M, Mediator; W, Moderator; SE, Standard Error; CI, Confidence Interval, BCa, Bias-Corrected and Accelerated.

**Table S24 Pathway from hyperactivity traits to depressive symptoms: Moderated mediation analysis with self-rumination and self-reflection**

| Path | IV  on M | | IV*W  on M | M  on DV | M*W  on DV | | IV on DV  (Direct) | | | |
| --- | --- | --- | --- | --- | --- | --- | --- | --- | --- | --- |
| Regression  coefficients  (standardized) | 0.11  (0.02)*** | | −0.02  (0.01) | 0.21 (0.02)*** | −0.04  (0.01)*** | | 0.23  (0.02)*** | | | |
|  | | Conditional indirect effects of IV on DV (standardized) | | | | | | | | |
| Moderator | | Condition | | Effect | | SE | | BCa of 95% CI | | |
| Self-reflection | |  | |  | |  | | | Lower | Upper |
|  |  | Low | | 0.03 † | | 0.01 | | | 0.02 | 0.05 |
|  |  | Middle | | 0.02 † | | 0.005 | | | 0.01 | 0.03 |
|  |  | High | | 0.02 † | | 0.005 | | | 0.01 | 0.03 |

Model. IV = Hyperactivity, M = Self-rumination, DV = Depression, W = Self-reflection

All values are reported after controlling for age, sex and Inattention scores. BCa represents the bias-corrected and accelerated 95% confidence interval of indirect effects. Intervals not containing 0 indicate a significant indirect effect. † denotes a significant indirect effect, as the 95% confidence interval does not include zero.

* *p* < 0.05, ** *p* < 0.01, *** *p* < 0.001, IV, Independent Variable; DV, Dependent Variable; M, Mediator; W, Moderator; SE, Standard Error; CI, Confidence Interval, BCa, Bias-Corrected and Accelerated.

These results indicate that self-rumination mediates the relationship between ADHD traits and depressive symptoms for both inattention and hyperactivity subtypes. The standardized coefficients showed that the indirect effect of inattention traits on depressive symptoms through self-rumination is stronger (Indirect Effect = 0.0590, BCa CI [0.0459, 0.0730]) compared to the indirect effect of hyperactivity traits (Indirect Effect = 0.0225, BCa CI [0.0134, 0.0327]). Furthermore, the protective effect of self-reflection against depressive symptoms is more pronounced in the relationship between inattention traits and depressive symptoms, mediated by self-rumination, than in the relationship between hyperactivity traits and depressive symptoms, as indicated by the significant interaction effect (Index of Moderated Mediation = −0.0064, BCa CI [−0.0104, −0.0030]). In contrast, the interaction was not significant for hyperactivity traits (Index of Moderated Mediation = −0.0028, BCa CI [−0.0088, 0.0031]). This suggests that while self-rumination consistently mediates the relationship between ADHD traits and depressive symptoms, the moderating role of self-reflection is more significant in the context of inattention traits.

**Methods S1: Detailed descriptions of questionnaires**

**Demographic questionnaire**

Participants provided answers related to their age and gender.

**Japanese version of the Adult ADHD Self-Report Scale**

In this study, we used the Adult ADHD Self-Report Scale (ASRS) to screen for potential ADHD and evaluate the severity of ADHD traits. Developed by the World Health Organization, ASRS is widely used in epidemiological studies on ADHD among adults.^1^ This six-item scale serves as a screening tool for adult ADHD (aged ≥ 18 years) based on the diagnostic criteria outlined in the Diagnostic and Statistical Manual of Mental Disorders, 4th edition, Text Revision (2000). This scale is standardized and well-validated for the assessment of current ADHD traits. Participants rate the frequency of particular symptoms over the past 6 months on a five-point Likert scale ranging from 0 (never) to 4 (very often). Total score is summed and can range from 0 to 24. Based on the classification criteria recommended by Takeda et al.,^2^ participants were classified as “possible ADHD” and “non-ADHD” if their ASRS score were ≥15 and < 15, respectively. The choice of 15 as the cutoff score was based on its validation in the Japanese population.^2^ The ASRS score was used as a continuous variable in our mediation analyses, with the sum of the first four items of ASRS relating to inattention as the “Inattention score” and the sum of the last two items relating to hyperactivity as the “Hyperactive score”.^3^

**Japanese version of Beck Depression Inventory-II**

The Beck Depression Inventory-II (BDI-II), which is a four-point Likert type comprising 21 questions, was developed by Beck et al. to measure the severity of depressive symptoms.^4^ The Japanese version was previously shown to be valid and reliable.^5^ Despite the ranges of depressive symptoms severity scores proposed by the original study,^4^ recent research has highlighted that different optimal cutoff points can be used for different populations.^6,7^ The BDI-II score was used as a continuous variable in the mediation analyses.

**Japanese version of Rumination-Reflection Questionnaire**

The Rumination–Reflection Questionnaire (RRQ) contains self-rumination and self-reflection subscales, and each subscale comprises 12 items rated on a five-point scale. Previous studies confirmed the good test-retest reliability and convergent validity of both this questionnaire^8^ and its Japanese version.^9^ Rumination and Reflection Scores, derived from the RRQ were utilized as continuous variables in our analyses. Scores for self-rumination or self-reflection ranged from 12 to 60, with higher scores indicating greater levels of each respective tendency.

**References S1**

1 Kessler, R. C. *et al.* The World Health Organization Adult ADHD Self-Report Scale (ASRS): a short screening scale for use in the general population. *Psychol Med* **35**, 245-256, doi:10.1017/s0033291704002892 (2005).

2 Takeda, T., Tsuji, Y. & Kurita, H. Psychometric properties of the Japanese version of the Adult Attention-deficit hyperactivity disorder (ADHD) Self-Report Scale (ASRS-J) and its short scale in accordance with DSM-5 diagnostic criteria. *Res Dev Disabil* **63**, 59-66, doi:10.1016/j.ridd.2017.02.011 (2017).

3 Das, D., Cherbuin, N., Butterworth, P., Anstey, K. J. & Easteal, S. A population-based study of attention deficit/hyperactivity disorder symptoms and associated impairment in middle-aged adults. *PLoS One* **7**, e31500, doi:10.1371/journal.pone.0031500 (2012).

4 Beck, A. T., Steer, R. A., Ball, R. & Ranieri, W. Comparison of Beck Depression Inventories -IA and -II in psychiatric outpatients. *J Pers Assess* **67**, 588-597, doi:10.1207/s15327752jpa6703_13 (1996).

5 Kojima, M. *et al.* Cross-cultural validation of the Beck Depression Inventory-II in Japan. *Psychiatry Res* **110**, 291-299, doi:10.1016/s0165-1781(02)00106-3 (2002).

6 Wang, Y. P. & Gorenstein, C. Psychometric properties of the Beck Depression Inventory-II: a comprehensive review. *Braz J Psychiatry* **35**, 416-431, doi:10.1590/1516-4446-2012-1048 (2013).

7 von Glischinski, M., von Brachel, R. & Hirschfeld, G. How depressed is "depressed"? A systematic review and diagnostic meta-analysis of optimal cut points for the Beck Depression Inventory revised (BDI-II). *Qual Life Res* **28**, 1111-1118, doi:10.1007/s11136-018-2050-x (2019).

8 Trapnell, P. D. & Campbell, J. D. Private self-consciousness and the five-factor model of personality: distinguishing rumination from reflection. *J Pers Soc Psychol* **76**, 284-304, doi:10.1037//0022-3514.76.2.284 (1999).

9 Takano, K. & Tanno, Y. Development of Japanese-version Rumination-Reflection Questionnaire. *The Japanese Journal of Personality* **16**, 259-261, doi:10.2132/personality.16.259 (2008).

**Methods S2: Bias mitigation strategies**

**Selection bias**

Participants were randomly selected from across Japan and stratified by district, gender, and age to ensure a representative sample. This stratification helped to mitigate selection bias by ensuring that the sample reflected the broader population.

**Information bias**

Standardized self-report scales, namely ASRS, BDI-II, and RRQ, were used to measure ADHD traits, depressive symptoms, and self-rumination/self-reflection tendencies, respectively. We provided all participants with the same instructions and information to ensure consistency in their responses, thereby reducing information bias.

**Reporting bias**

To encourage honest reporting and mitigate reporting bias, participants were assured of the anonymity of their responses.

**Confounding bias**

Key confounders, such as age and gender, were included in the statistical models to control their potential impact on our results. Adjustments for these variables were made in the analyses to mitigate confounding bias.

**Methods S3: Linear regression for mediation analysis**

We evaluated the predictive capacity of the independent variable and its interaction with the mediator on the dependent variable using multiple regression analyses. Multicollinearity was assessed with Variance Inflation Factor (VIF) and tolerance values, with values of VIF < 5 and tolerance > 0.2 indicating its absence,^10^ thus ensuring the robustness of our model. The overall significance of the regression models was determined using the F-statistic. A significant F value indicates that the model with predictors fits the data better than a null model, thereby confirming the reliability of the regression analysis.

**References S2**

10 Kim, J. H. Multicollinearity and misleading statistical results. *Korean J Anesthesiol* **72**, 558-569, doi:10.4097/kja.19087 (2019).

**Figure S2: Schematic diagrams of mediation, moderation, and moderated mediation analyses**

**Figure S2**


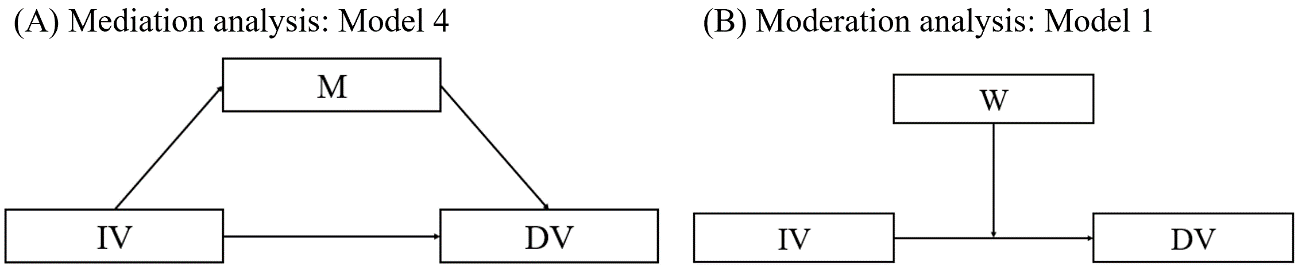


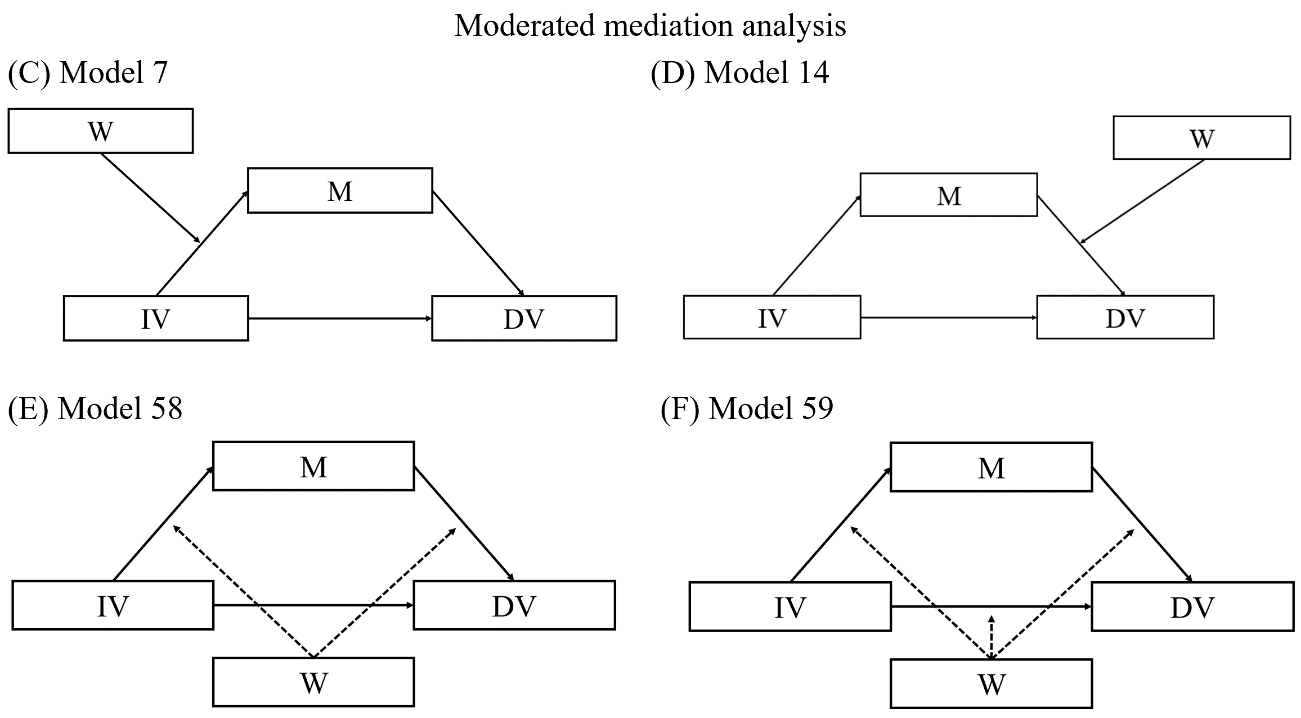


(A) Mediation analysis (Model 4): Analyzes the mediating effect of M on the relationship between IV and DV.

(B) Moderation analysis (Model 1): Evaluates how W moderates the relationship between IV and DV.

Moderated mediation analysis:

(C) Model 7: Examines if W moderates the effect of IV on M.

(D) Model 14: Investigates if W moderates the effect of M on DV.

(E) Model 58: Tests the moderating effect of IV on all paths in the mediation model.

(F) Model 59: Analyzes the moderating effect of W on the direct and indirect paths.

IV, Independent Variable; DV, Dependent Variable; M, Mediator; W, Moderator

**Methods S4: Mediation and moderation analyses to evaluate specific relationships within two triads**

**1. ADHD traits, self-rumination, and self-reflection**

- Mediating and moderating effects of self-reflection on the relationship between ADHD traits and self-rumination.

- Mediating and moderating effects of self-rumination on the relationship between ADHD traits and self-reflection.

**2. Self-rumination, self-reflection, and depressive symptoms**

-Mediating and moderating effects of self-reflection on the relationship between self-rumination and depressive symptoms.

- Mediating and moderating effects of self-rumination on the relationship between self-reflection and depressive symptoms.

**Methods S5: Moderated mediation analysis**

The moderated mediation hypothesis was particularly examined using the PROCESS macro (v.4.2), Models 7, 14, 58, or 59. This analysis aimed to estimate the moderated mediation effects of all variables, considering different paths and interactions:

Model 7 (Figure S2C):

Assesses the moderator’s effect on the relationship between ADHD traits and the mediator.

Model 14 (Figure S2D):

Examines the moderator’s effect on the relationship between the mediator and depressive symptoms.

Model 58 (Figure S2E):

Evaluates the moderator’s influence on both paths from ADHD traits to the mediator and from the mediator to depressive symptoms.

Model 59 (Figure S2F):

Investigates the moderator’s impact on the direct and indirect effects of ADHD traits on depressive symptoms.

We tested both cases, i.e., where self-rumination or self-reflection acted as either the moderator or the mediator. Standardized regression coefficients were computed along with unstandardized regression ones to compare the effect sizes of each variable in these models, with standardization applied to all variables. Gender, considered as a covariate, was set as a categorical variable and was addressed by employing a dummy variable, assigning a value of 0 for men and 1 for women.
